# Cytocompatibility of stabilized black phosphorus nanosheets tailored by directly conjugated polymeric micelles for human breast cancer therapy

**DOI:** 10.1038/s41598-021-88791-7

**Published:** 2021-04-29

**Authors:** M. Biedulska, P. Jakóbczyk, M. Sosnowska, B. Dec, A. Muchlińska, A. J. Zaczek, D. Nidzworski, R. Bogdanowicz

**Affiliations:** 1 Institute of Biotechnology and Molecular Medicine, Trzy Lipy 3, 80-172 Gdańsk, Poland; 2 Faculty of Electronics, Telecommunications and Informatics, Gdańsk University of Technology, Narutowicza 11/12, 80-233 Gdańsk, Poland; 3 Laboratory of Translational Oncology, Intercollegiate Faculty of Biotechnology, Medical University of Gdansk, Debinki 1, 80-211 Gdansk, Poland; 4 Department of Recombinant Vaccine, Intercollegiate Faculty of Biotechnology, University of Gdansk and Medical University of Gdansk, Kladki 24, 80-822 Gdansk, Poland

**Keywords:** Biochemistry, Biotechnology, Cancer, Materials science

## Abstract

The novel procedure of few-layer black phosphorus (FLBP) stabilization and functionalisation was here proposed. The cationic polymer PLL and non-ionic PEG have been involved into encapsulation of FLBP to allow sufficient time for further nanofabrication process and overcome environmental degradation. Two different spacer chemistry was designed to bind polymers to tumor-homing peptides. The efficiency of functionalisation was examined by RP-HPLC, microscopic (TEM and SEM) and spectroscopic (FT-IR and Raman) techniques as well supported by ab-initio modelling. The cell and dose dependent cytotoxicity of FLBP and its bioconjugates was evaluated against HB2, MCF-7 and MDA-MB-231 cell lines. Functionalisation allowed not only for improvement of environmental stability, but also enhances therapeutic effect by abolished the cytotoxicity of FLBP against HB2 cell line. Moreover, modification of FLBP with PLL caused increase of selectivity against highly aggressive breast cancer cell lines. Results indicate the future prospect application of black phosphorus nanosheets as nanocarrier, considering its unique features synergistically with conjugated polymeric micelles.

## Introduction

Since graphene was isolated by Novoselov et al*.*^1^, two-dimensional (2D) few-layered materials have been attracting increasing attention due to their unique electronic^2^, optical^3^, catalytic^4^, electrochemical^5^, and mechanical^6^ properties in comparison to their multilayered bulk counterparts. The family of 2D materials is a large group of nanomaterials, such as graphene, transition metal dichalcogenides (molybdenum disulfide), MXene^7^, and phosphorene, which was discovered in 2014. Phosphorene shows properties such as anisotropy, high carrier mobility, mechanical flexibility, and a tunable bandgap from 0.3 eV (bulk) to 2.0 eV (single layer)^8^. For biomedical applications, its strong absorption in the ultraviolet (UV) and near-infrared (NIR) regions^9,10^, biocompatibility, and great biodegradability in the physiological environment are especially important. These properties of phosphorene have attracted special attention for clinical applications in biomedicine such as phototherapy^11,12^, drug delivery^13^, biosensing^14,15^, and theranostics^10,16^.

Although studies of biomedical applications of FLBP have made a lot of progress, there are still some problems that need to be solved before its clinical transition. Various factor such as size, concentration and test cell line have a great impact on toxicity and treatment effects, it is very important to find an effective method to fabricate FLBP with uniform size and high production. It was reported that BP nanosheets does not cause haemolysis when cultured with red blood cells. BP does not induce significant changes in the liver or kidney functions comparing with untreated healthy rats^17^. Similar experiments were performed in mice where no significant inflammation and infection was induced by BP and after 30 days all blood biochemical parameters were normal^18,19^. Another remarkable but still underexplored feature of phosphorene is it potential as a carrier for biological and biomedical molecules. Surface functionalisation or conjugation is commonly used to increase stability, improve drug loading and cellular uptake, actively target cancer cells and tumors. To environmental stability of phosphorene is also concern and specialized coatings are necessary to ensure stable performance of the sensor. In this regard, the number functionalisation with other nanoparticulate system or polymers (poly(ethylene glycol) (PEG), poly-l-lysine (PLL), Poly(lactic-co-glycolic acid) (PLGA), polysialic acid or glycolic acid) have been developed^20,21^. The fabrication of BP nanoplatform with polymers could be efficient route to achieve stability of nanomaterial and enhancement of its properties. On the one hand, the involvement of non-ionic, flexible, hydrophilic and biocompatible PEG polymer allowed to resolve the problem with non-specific absorption on the nanomaterial surface^22–26^. While, application of ionic PLL polymer in functionalisation of nanosheet surface may be a new strategy to overcome drug resistance related to deficient transport^27,28^. The polycationic nature of poly-l-lysine enhanced the interaction with plasmid DNA and ensured the protection of biomolecules from nuclease degradation^29,30^. These functionalised strategy allow to applied BP in different application area, considering unique features of nanosheets itself or utilizing advantage of synergistic effect of applied components. Constructing such a nanoplatform not only enables encapsulation of specific recognition ligands, but can result in targeted drug delivery to the tumor cell^31–33^.

Peptides may offer new therapeutic opportunities due to low immunogenicity, excellent tissue penetration and low production cost^34,35^. Specific targeting of therapeutic moieties to cancer tissue may be a valuable option, especially when local treatment is not possible. The positively charged amino acid residues have great potential as an active targeting ligand^36–38^. Peptides rich in KRK motif could promote membrane fusion through electrostatic interaction with lipid and enable the interaction with negatively charged DNA^39^. This short sequence was uses because it possesses ability to accumulation both on the surface of tumor vessels and within tumor tissue after intravenous injection^40,41^.

Next, peptides which can be selectively delivered to tumors are very attractive biomolecules for the prevention and treatment of cancer. The arginine-glycine-aspartic acid (RGD) sequence plays a crucial role in cellular adhesion of extracellular fibronectins^42^. RGD motif exhibit a strong affinity and selectivity to alpha v beta 3 integrin on the cell surface and enter the cytoplasm receptor-mediated endocytosis^43^. Within the present work, RGD sequence was used due to the fact that RGD triad is a typical cell-binding domain in order to effective targeting of endothelial cells in tumor vasculature and suppressing angiogenesis and tumor growth. Many research effort have been paid to the determination of therapeutic efficiency and selective tumor targeting ability of black phosphorus. Unfortunately, studies describe the difference in efficiency of the applied linker in delivery systems are still missing. Drug delivery studies with different structural linkers are often performed in different tumor models with a wide dose range, which makes them difficult to compare. Therefore extensive investigations about BP and its bioconjugates various applications as well as critical evaluation on their toxicity assessment are very crucial before introducing it into practical area.

The novel strategies of FLBP nanosheets functionalisation are presented in the paper allowing for enhancement of its environmental stability against air or aqueous solvents. We applied two different strategies of bioligands grafting studying influence of ionic character, surface conformation and biological activity of poly-l-lysine (PLL) and poly-ethylene glycol (PEG), in order to overcome FLBP nanosheets degradation. To the best of our knowledge, the functionalisation of nanosheet incorporating bioligands—tumor-homing peptides with KRK and RGD motifs as a potential drugs in breast cancer therapy were not reported in the literature up to date. The toxicity investigation of FLBP itself and its modifications were carried out in two model of breast cancer lines MCF-7 (luminal A subtype of breast cancer) and MDA-MB-231 (highly aggressive, triple-negative breast cancer).

## Materials and methods

### Materials

The black phosphorus was purchased from Smart Elements. N,N-Dimethylformamide (DMF) was collected from Sigma Aldrich. All reagents were analytical grade and were used without further purification. The purge gas (argon) used for the liquid exfoliation process was purchased from Air Liquid and had the highest purity class. The Fmoc-protected amino acids for the synthesis of the peptides were purchased from Iris Biotech GmbH (Marktredwitz, Germany). The TentaGel S PHB resin was acquired from Rapp Polymere GmbH (Tuebingen, Germany). The *N,N*-Diisopropylethylamine (DIPEA), 2-(1H-benzotriazole-1-yl)-1,1,3,3-tetramethylaminium tetrafluoroborate (TBTU), triisopropylsilane (TIS), N-hydroxysuccinimide (NHS), (1-ethyl-3-(3-dimethylaminopropyl)carbodiimide hydrochloride (EDC) and poly-l-lysine hydrobromide (30,000–70,000) 1-methylimidazol (Melm) and 2,4,6-mesitylene-sulfonyl-3-nitro-1,2,4-triazol (MSNT Novabiochem^®^) were received from Sigma Aldrich Company. The Hydroxy-poly(ethylene glycol) amine NH_2_-PEG-OH·HCl (MW 2000) was obtained from JenKem Technology USA. The peptide synthesis-grade *N,N*-dimethylformamide was purchased from Acros Organics. The Acetonitrile HPLC gradient grade and trifluoroacetic acid for the HPLC were purchased from Alfa Aesar. Doubly distilled water (Hydrolab-Reference purified) with conductivity not exceeding 0.05 µS cm^−1^ was used. All other chemicals used in this study were analytical reagent grade and used without further purification.

### Parameters of FLBP properties ab-initio study

The few-layer black phosphorus model was prepared in the Quantum ATK software from Synopsys^44^ in order to understand how surface oxidation influences adsorption of linker molecules. The created supercell was constructed using the (C m c a) space group. The Center Orthorhombic Bravais lattice was used in all slabs. The properties were calculated with Generalized Gradient Approximation using Perdew-Burke-Ernzerhof^45^ (GGA-PBE). The description of the Van der Waals forces was performed with the Grimme-D2 corrections method. The Fritz-Haber Institute (FHI) pseudopotential code with Double Zeta Polarized basis set was used. The reference point for the simulation parameters was bulk phosphorus and was calculated with a bandgap of 0.284 eV, which is in great accordance to the experimental value 0.3 eV^46,47^. Surface configurations were modelled with the exact same parameters as bulk phosphorene including a 30 Å vacuum in the Y-direction in order to create the surface model.

### Liquid exfoliation of black phosphorus

**Figure 1:**
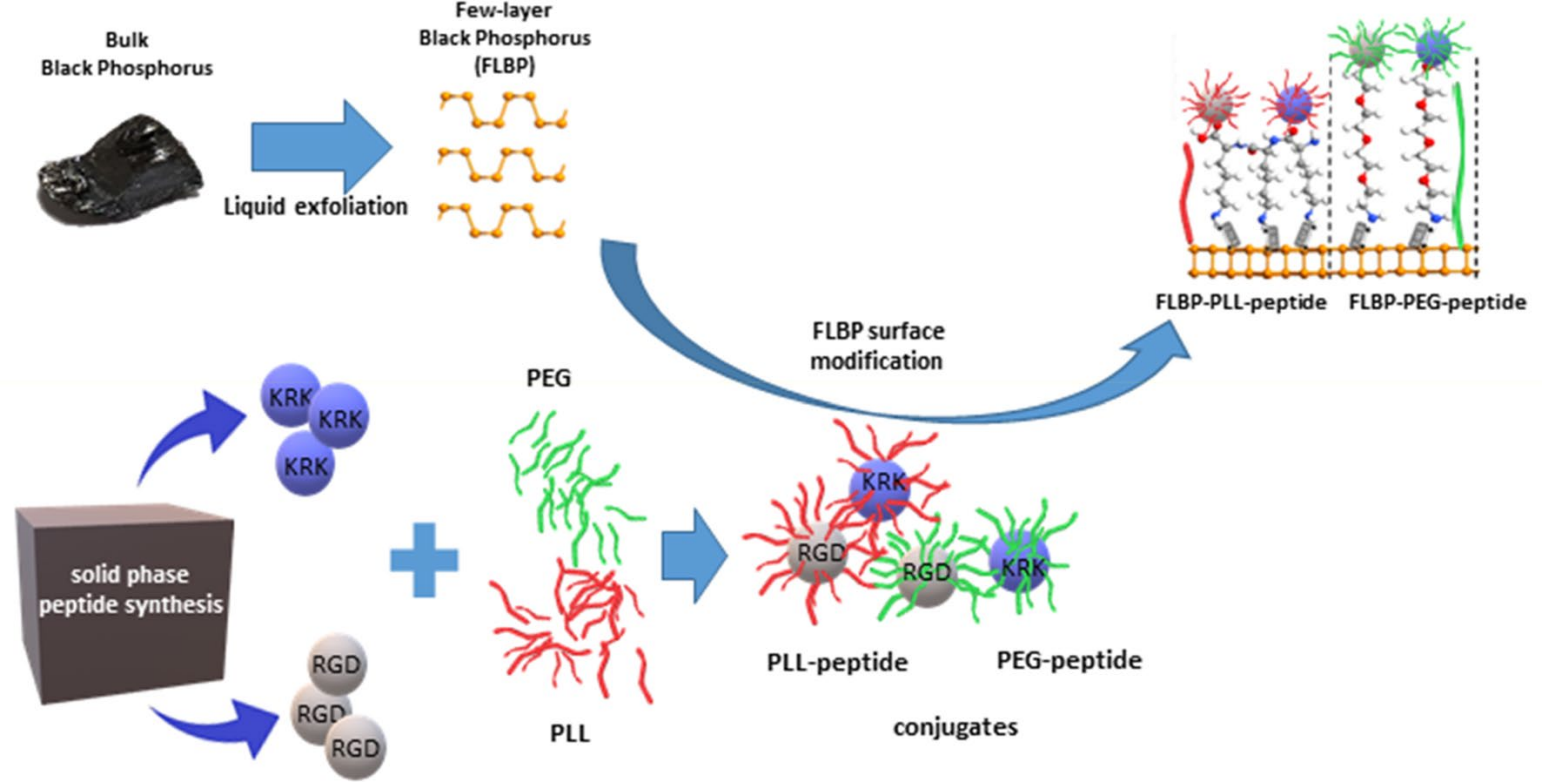
Scheme processes to yield FLBP-linker-peptide conjugates [partially drawn with QuantumATK Q-2019.12 (https://www.synopsys.com/silicon/quantumatk/resources/release-notes.html)].

Few-layer black phosphorus (FLBP) was prepared from pre-ground black phosphorus (BP) crystals (60–70 mg) dispersed in deoxygenated anhydrous dimethylformamide (7 mL). During the liquid exfoliation process, the sample was kept in an ice bath, at a temperature from 0 to 3 °C, under a stream of argon using a horn probe ultrasonicator (Bandelin Sonopuls HD2200, 20 kHz). The sonication power and time amplitudes were 40 W and 0.5 s/0.5 s, respectively. The FLBP suspension in DMF was processed for 120 min. and afterwards was centrifuged at 6000 rpm for 5 min to remove the residual unexfoliated particles, yielding supernatant. Next, the exfoliation was repeated for the unexfoliated BP for another 2 h. After this step, the centrifugation of the re-exfoliated suspension was repeated. Finally, the FLBP with a concentration of 1.2–1.5 mg per solvent millilitre and zeta potential close to 0 mV was used for the reaction as shown in Fig. 1.

### Synthesis and purification of tumor-homing peptides

The H-SPRGDGG-OH (RGD) and H-SPKRKGG-OH (KRK) peptides were synthesised by standard solid phase 9-fluorenylmethoxycarbonyl (Fmoc) chemistry^29,30^ using a Magnum Nova 10 microwave reactor (Ertec, Poland). Tentagel S PHB resin with 0.27 mmol g^−1^ capacity was applied to produce C- terminal COOH peptides. Anchoring the first Fmoc-amino acids to the hydroxyl solid support was performed by esterification of the carboxylic acid with 2,4,6-mesitylene-sulfonyl-3-nitro-1,2,4-triazol (MSNT) in the presence of 1-methylimidazol (Melm)^30^. The remaining coupling steps were carried out at threefold excess over the resin capacity. Before each coupling cycle, the pre-activation step of Fmoc-AA was performed by the addition of 2.85 equivalents of *N,N*-Diisopropylethylamine (DIPEA) and 6.00 equivalents of 2-(1H-benzotriazole-1-yl)-1,1,3,3-tetramethylaminium tetrafluoroborate (TBTU) to the amino acid solution in DMF, respectively. Microwave-assisted coupling reactions were repeated twice under constant conditions (7 W of power, 60 °C, 5 min). The Fmoc group deprotection involved 20% piperidine solution in DMF and was carried out under 5 W of microwave irradiation power twice for 4 min at 60 °C. During microwave-assisted SPPS, continuous stirring was maintained by using a nitrogen stream. Cleavage of the desired peptide from the resin with simultaneous side chain protecting group removal were performed by treatment with a mixture containing 95.00% trifluoroacetic acid (TFA), 2.50% triisopropylsilane (TIS) and 2.50% water for 2 h at room temperature. After cleavage from the resin, the peptides were precipitated in cold, anhydrous ethyl ether and separated from soluble non-peptide substances by centrifugation (4 °C, 4000 rpm, 3 × 15 min.). The obtained pellets were dissolved in deionised water and the obtained solutions were lyophilised. The mass analysis of crude peptides was performed by Matrix-Assisted Laser Desorption/Ionisation Time of Flight Mass Spectrometry MALDI-TOF MS (Bruker, Germany) with α-Cyano-4-hydroxycinnamic acid (CCA) and 2,5-Dihydroxybenzoic acid (DHB) as a matrix. The crude products were purified by a HPLC system (Shimadzu Prominence Modular HPLC SPD-20A UV–Vis detector) using a semi-preparative reverse phase Phenomenex Gemini C18 column (250 mm × 30 mm, 110 Å, 5 µm). A linear gradient elution was applied from 5 to 20% of solvent B at a flow rate of 18 mL min^−1^ for 60 min. The gradient used water (A) and acetonitrile (B) as eluants containing 0.10% TFA. The UV detection was carried out at λ_1_ = 224 nm and λ_2_ = 254 nm. The purity of the desired peptides was evaluated using an analytical reverse-phase HPLC Shimadzu system (Shimadzu Prominence-i LC-2030C) with a Cosmosil C18 column (4.60 mm × 250 mm, 90 Å, 5 µm) using the linear gradient method from 0 to 100% solvent B for 30 min at a flow rate of 1.50 mL min^−1^ with UV detection at λ1 = 224 nm and λ2 = 254 nm. Fractions containing above 98% (by analytical HPLC) of the expected peptide were pooled and lyophilised. MALDI TOF mass analysis of the purified peptides confirmed the formation of the desired products.

### Functionalisation procedure of FLBP

#### Preparation of FLBP-poly-l-lysine-peptides

Preparation of the FLBP-PLL-peptide structure was performed in two stages. In the first part peptide attach to the linker via EDC/NHS mediation , which provides a covalent amide bond between the carboxyl group of the linker (PLL) and an N-terminal amine moiety of the peptide without the addition of a spacer. Briefly, 38.10 mg of N-hydroxysuccinimide (NHS), 63.70 mg of 1-Ethyl-3-(3-dimethylaminopropyl) carbodiimide hydrochloride EDC, and 1.60 mg of PLL∙HBr were dissolved in 1.50 mL of ultrapure water. For linkage, 2 mL of 12 mM aqueous solution of RGD peptide was added to the reaction mixture. Poly-l-lysine modification was carried out under stirring continuous for 24 h at room temperature. The progress of the reaction was monitored by Reverse Phase High Performance Liquid Chromatography (RP-HPLC). Afterwards, the modified linker was centrifuged (9000 rpm, 4 °C) to remove unconjugated peptide. After centrifugation, the obtained linkage product was lyophilised.

In the next step, the conjugation of PLL-peptide with FLBP was carried out. The FLBP was resuspended in 2 mL of ultrapure water and then 2 mL the desired PLL-RGD aqueous solution was added. The reaction mixture was placed in a round-bottom flask in an ice bath (0 °C) and continuous stirring was maintained for 1 h through an argon stream. After this period, the sample was incubated for 24 h at 4 °C. Afterwards, the obtained reaction mixture was centrifuged at 9000 rpm for 30 min. The obtained black powder was lyophilised. The same synthetic protocol was applied for the conjugation of FLBP with PLL-KRK.

In order to verify the surface modification of the nanoparticles with the linker-peptides, the prepared samples were lyophilised via an Alpha 2-4 LSCbasic Freeze Dryer Martin Christ (0.070 Mbar Vacuum, T = − 80 °C), and then subjected to Scanning Electron Microscopy, Transmission Electron Microscopy analysis and spectroscopic assay including Raman and FT-IR spectra to confirm successful conjugation of the nanoparticles with the peptides.

### Preparation of FLBP-PEG-peptides

The conjunction between the few-layer black phosphorus and the PEG-peptide couple can only take place after prior activation of the hydroxyl groups in the PEG and then pegylation of a peptide. In order to covalently attach the peptide molecule to hydroxy-terminated PEG (H_2_N-PEG-OH), the chloroformate activation strategy of hydroxyl groups on end-functionalised PEG was used. The reaction between the peptide and the H_2_N-PEG-OH takes place between the activated H_2_N-PEG-*p*-nitrophenyl carbonate molecule which is reactive towards the α-amino group of the peptide. For this purpose, the linear H_2_N-PEG-OH (Mw = 2000 g mol^−1^) 0.20 g, 0.10 mmol was dissolved in 5 mL of dry toluene. To this solution, 4-nitrophenyl chloroformate (60.45 mg, 0.30 mmol) and ethylene chloride (2 mL) were added and the pH was brought to 8.00 with TEA (120 µL, 0.86 µmol). The obtained H_2_N-PEG-*p*-nitrophenyl carbonate was precipitated with diethyl ether (20 mL) and the resulting product was purified by crystallisation from ethyl acetate.

Afterwards, the H-SPRGDGG-OH (RGD) peptide (15.90 mg, 24.60 µmol) was dissolved in 2.50 mL of H_2_O/CH_3_CN (90:1) in the presence of TEA (3.4 µL, 24.20 µmol) and previously obtained H_2_N-PEG-*p*-nitrophenyl carbonate (10.50 mg, 4.88 µmol) was added. The aqueous solution was kept at room temperature and stirred for 48 h. The pH of the solution was brought to a pH 3.00 and the product was extracted into chloroform (five times, 50 mL each), concentrated and precipitated with diethyl ether.

The H-SPKRKGG-OH (KRK) peptide (20.10 mg, 27.58 µmol) was dissolved in 2.50 mL of H_2_O/CH_3_CN (90:1) in the presence of TEA (3.80 µL, 27.30 µmol) and previously obtained H_2_N-PEG-*p*-nitrophenyl carbonate (10.50 mg, 4.88 µmol) was added. The aqueous solution was kept at room temperature and stirred for 48 h. The pH of the solution was brought to 3.00 and the product was extracted into chloroform (five times, 50 mL each), concentrated and precipitated with diethyl ether. The resultant conjugates were characterised by HPLC chromatography using a Cosmosil C18 (4.60 × 250 mm, particle size 5 µm) reverse phase column. The gradient used water (A) and acetonitrile (B) as eluants containing both 0.10% TFA was from 0 to 100% B for 30 min, the flow rate was 1.50 mL min^−1^ with UV detection at λ_1_ = 214 nm and. λ_2_ = 254 nm. The masses of the obtained PEG-peptide conjugates were confirmed by MS analysis. MALDI-TOF mass spectrometry was carried out on a SCIEX TOF/TOF™ 5800 System works using the MALDI ionisation technique (Matrix Assisted Laser Desorption Ionisation). The matrix used in the MALDI-TOF mass spectrometry was 2,5-dihydroxybenzoic acid (DHB).

FLBP was modified with H_2_N-PEG-RGD-OH and H_2_N-PEG-KRK-OH conjugates, respectively. FLBP gathered by liquid exfoliation (0.26 mg) was re-suspended in 4 mL of aqueous solution H_2_N-PEG-RGD-OH conjugate (2.50 mg mL^−1^). After stirring for 24 h, the obtained H_2_N-PEG-RGD-OH-loaded FLBP nanoparticles (FLBP-PEG-RGD) were centrifuged and washed with deoxygenated water. The same protocol was applied for modification of FLBP with H_2_N-PEG-KRK-OH peptide and obtained H_2_N-PEG-KRK-OH-loaded FLBP nanoparticles (FLBP-PEG-KRK).

In order to verify the modification of the FLBP surface with the PEG-peptide conjugates, the prepared samples were lyophilised and then subjected to Transmission Electron Microscopy, Scanning Electron Microscopy analysis and spectroscopic assay including Raman and FT-IR spectra.

### In vitro cytotoxicity

The in vitro cytotoxicity of the PEG, KRK, RGD, PLL and FLBP, FLBP-PEG-RGD, FLBP-PEG-KRK, FLBP-PLL-RGD and FLBP-PLL-KRK was assessed in MCF-7, MDA-MB-231 (human breast adenocarcinoma cell lines) and HB2 (human mammary luminal epithelial cell line) cells by a standard methyl thiazolyl tetrazolium (MTT) assay. MCF-7 and MDA-MB-231 were cultured in DMEM supplemented with 10% foetal bovine serum (FBS) and 1% penicillin–streptomycin solution. HB2 was cultured in DMEM supplemented with 10% foetal bovine serum (FBS), 1% penicillin–streptomycin solution, 5 μg mL^−1^ hydrocortisone, and 5 μg mL^−1^ insulin. The cells were routinely tested for mycoplasma contamination. All of the media and their supplements were from Sigma-Aldrich (Saint Louis, MO, USA) or HyClone (GE Healthcare, Chicago, IL, USA). 7 × 10^3^ viable cells per well were seeded in 96-well plates, and allowed to adhere overnight at 37 °C. After 24 h of incubation, culture medium was replaced with fresh medium and the cells were treated with different concentrations of PEG, KRK, RGD, PLL, FLBP, FLBP-PEG-RGD, FLBP-PEG-KRK, FLBP-PLL-RGD, or FLBP-PLL-KRK (0, 0.80, 4, 20 μg mL^−1^) for 72 h. Subsequently 20 μl of MTT working solution (5 mg mL^−1^) was added to each well and the plate was incubated for 1 h at 37 °C. The medium was then aspirated, and the formed formazan crystals were solubilised by adding 100 μl of DMSO (dimethyl sulfoxide). The absorbance was measured at 570 nm using a microplate reader (Synergy™ H1, BioTek, USA). The cell viability was calculated using the following formula: cell viability = Asample/Acontrol × 100%, where A is the absorbance at 570 nm. Data are presented as means ± SD from at least three independent experiments. Comparative data were analysed with the unpaired Student’s t-test using the IBM SPSS statistics software.

### Characterisation techniques

The Raman spectra were collected on a micro-Raman spectrometer (InVia, Renishaw, United Kingdom) with a 532 excitation laser (Ar ion laser), and the wavenumber was in the range of 300–600 cm^−1^. For the identification of functional groups in the obtained synthesis products, the FT-IR spectra were produced with a Bruker IFS66 spectrometer. The compounds were prepared for spectral analysis by using potassium bromide (KBr) pellets. The scanning electron microscope (SEM) (FEI Quanta FEG 250) allows the the evolution of the surface morphology to be observed. A 10 kV beam accelerating voltage was used with a Secondary Electron-Everhart–Thornley Detector (SE-ETD) working in a high vacuum mode pressure of 10^–4^ Pa to record high-resolution, high-magnification images of backscattered electrons emitted from the samples’ surfaces.

The morphology of the exfoliated and functionalised FLBPs were examined by a Tecnai G2 Spirit BioTWIN (FEI) transmission electron microscope (TEM) operated at an accelerating voltage of 120 kV to observe changes of the surface as a consequence of nanoparticle conjugation with the peptides. Briefly, 10 µL of each sample was drop cast onto a 300 mesh Copper TEM grid coated with Formvar Carbon film (EM Resolution). After deposition, the excess of solvent was filtered. Grids were rapidly transferred to the microscope with a minimal amount of light exposure to minimise the photo-oxidative degradation.

## Results and discussion

### Tumor-homing peptide characterisation

The crude peptides were purified by semi-preparative RP-HPLC on a C18 Column. The degree of purity for a single eluted peak of H-SPRGDGG-OH and H-SPKRKGG-OH peptides was always above 98% according to the analytical HPLC with a retention time at 4.757 min. and 4.595 min., respectively. The molecular weight of pure peptides was determined using MALDI TOF mass spectrometry. The observed pseudomolecular ion [M+H]^+^ at 645.70 Da in the mass spectrum of the RGD and 729.20 Da in the case of the KRK were consistent with the calculated molecular weights of 644.60 Da and 728.80 Da, respectively. The designed and synthesised peptides possessed a free carboxyl group on the *C*-terminus in order to further conjugate with the poly-l-lysine and OH-PEG-NH_2_ linkers.

### Characterisation of black phosphorus after attachment of linker-peptide fragments

In the presented studies, two-step bioconjugation of the linker-peptide hybrid to the black phosphorus nanoparticles was used. The first step involved covalent bioconjugation of the peptide with the poly-l-lysine (PLL) or poly(ethylene glycol) (PEG) linker. However, in the second step, the non-covalent immobilisation of the obtained hybrid (PLL-peptide or PEG-peptide) to the FLBP surface was applied. The RGD and KRK peptides were utilised as functional ligands to be conjugated to the surface of few-layer black phosphorus nanoparticles.

#### FLBP functionalisation with poly-l-lysine-peptide conjugate

**Figure 2:**
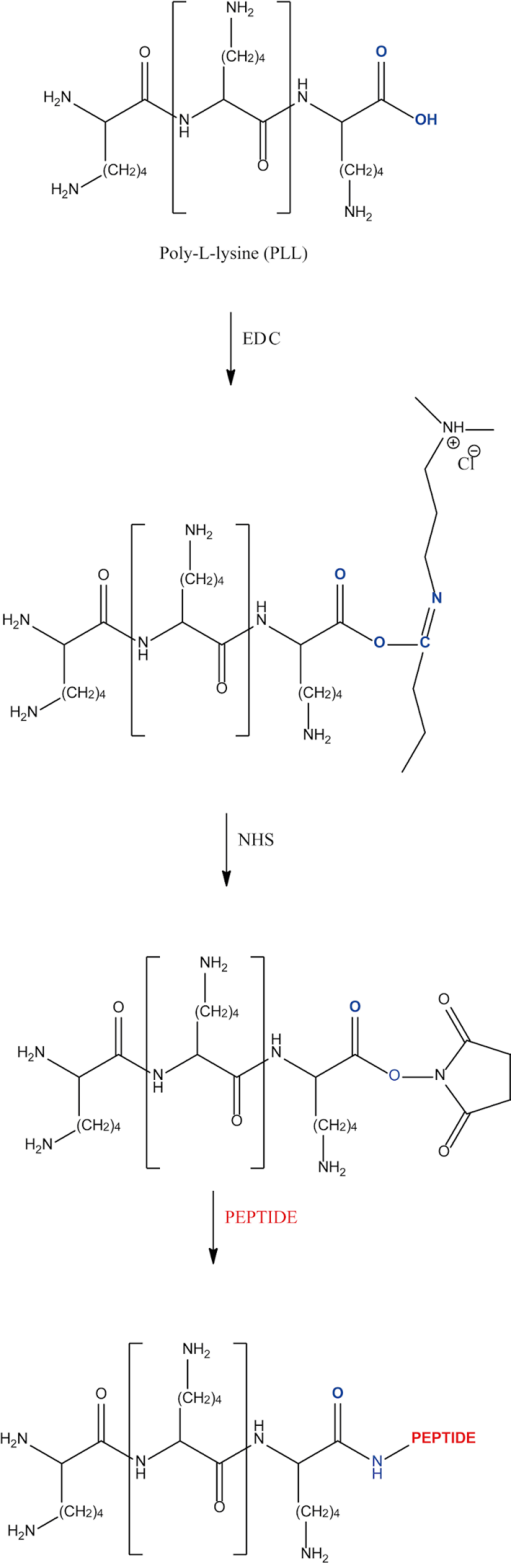
Linker (poly-l-lysine) is bound to a peptide through a EDC/NHS carbodiimide coupling reaction. Active ester was created from a carboxyl group of PLL. Primary amine group of selected peptide reacts and covalently binds to reactive ester.

The PLL linker was used to coat and protect the surface of the FLBP nanoparticles from degradation and to decrease non-specific interactions. The synthesised peptides were functionalised to the linker through *N*-terminal covalent conjugation using the carbodiimide coupling reaction aiming at precise dual targetting efficiency. The carboxylic group of the PLL could be reacted with the primary amine group of the obtained peptides by a condensation reaction to yield amide bonds. It should be noted that the carboxyl moiety must be chemically modified to achieve this goal. For this reason, EDC/NHS chemistry was applied to form a covalent amide bond without any addition of a spacer^30^. Briefly, the carboxylic group of the poly-l-lysine reacted with (1-ethyl-3-(3-dimethylaminopropyl)carbodiimide hydrochloride (EDC) to form an O-acylisourea intermediate which is unstable in an aqueous solution. Therefore, in a covalent immobilisation protocol, N-hydroxysuccinimide (NHS) was involved to increase the efficiency or formation of a dry-stable, amine reactive by-product. After formation of a reactive NHS ester from the carboxylic group, which is considerably more stable than the O-acylisourea intermediate, the activated group could be efficiently coupled to primary amines at a physiologic pH^30^. The *N*-terminal primary amine group of the peptide covalently binds to the reactive ester to form a peptide bond without the addition of a spacer (Fig. 2). As a consequence, covalent bioconjugation provides stronger bond formation and a more stable hybrid than physisorption.

**Figure 3:**
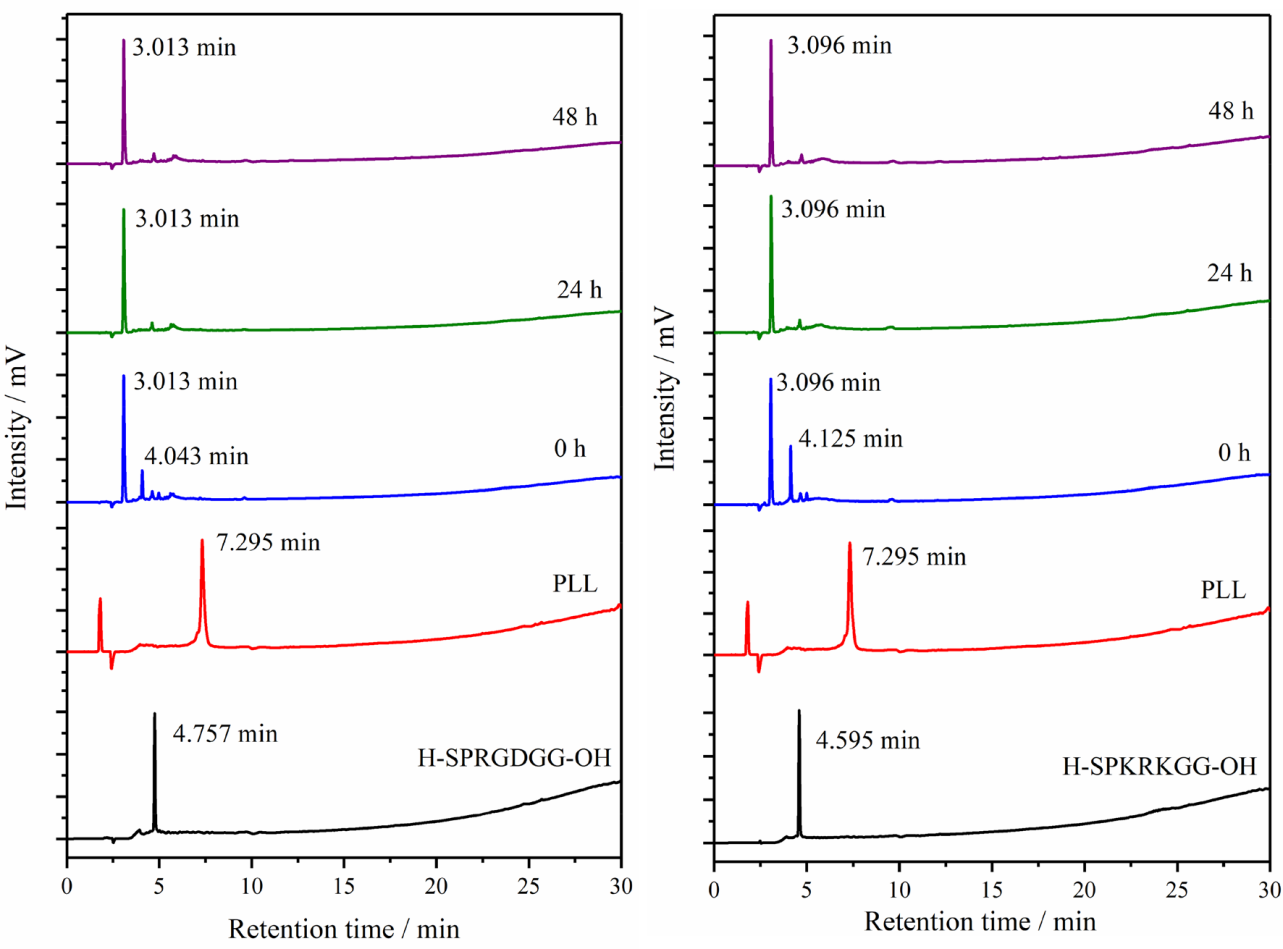
Covalent bioconjugation of PLL linker with RGD and KRK peptide reaction progress monitored by Reversed Phase High Performance Liquid Chromatography. RP-HPLC conditions: HPLC Shimadzu system (Shimadzu Prominence-i LC-2030C) with Cosmosil C18 column (4.60 mm × 250 mm, 90 Å, 5 µm) using linear gradient method from 0 to 100% solvent B for 30 min at flow rate of 1.50 mL min^−1^ with UV detection at λ_1_ = 224 nm and λ_2_ = 254 nm.

The progress of covalent immobilization of poly-l-lysine with the RGD peptide was monitored by the Reversed Phase High Performance Liquid Chromatographic method with UV detection. The RP-HPLC chromatograms of the substrates showed one sharp peak each with the following retention times: RGD: 4.757 min.; KRK: 4.595 min. and PLL: 7.295 min, respectively (Fig. 3).

The RP-HPLC reaction monitoring started immediately after adding the linker with coupling agents to the selected peptide solution. The presented approach focused on a fast analysis run to observe any changes during the conjugation reaction progress. As can be seen in Fig. 3, after the addition of the PLL with the crosslinker bioconjugate reagents (EDC and NHS) to the peptide solution, the signals corresponding to the substrate disappeared. At the same time, two new peaks appeared in the reaction mixtures’ HPLC chromatograms with retention times in the range 3.013–3.096 and 4.043–4.125 min, respectively. After 24 h, the HPLC chromatogram was recorded to evaluated the progress of bioconjugation. One major, sharp peak with a retention time of 3.013 min was observed, which corresponded to the formation of the expected PLL-RGD hybrid product. The shape of the reaction mixture’s HPLC chromatogram remained essentially constant thereafter for 48 h. Therefore, the reaction was considered to be completed. The efficiency of the poly-l-lysine coupling reaction with the PLL-KRK-OH peptide was evaluated in the same manner.

The qualitative RP-HPLC analysis presented in Fig. 3 revealed that the expected product was obtained after 24 h. The chromatogram is characterised by the presence of one sharp peak associated with the formation of the PLL-KRK conjugate with a retention time of 3.096 min. As in the case of the PLL-RGD hybrid, no addition signals appeared after 48 h of reaction.

A simple, noncovalent modification strategy was applied to obtained poly-l-lysine—peptide conjugates with few-layer black phosphorus (FLBP-PLL-peptide). The bioconjugation involved the combination of hydrophobic and electrostatic interactions of the PLL-peptide fragments and the FLBP surface. Briefly, poly-l-lysine belongs to a cationic polymer group with an isoelectric point around 9.50^48^. The acid–base equilibria studies revealed that three pKa constant values could be determined. In a strong acidic environment, the first pKa_1_ value equals 2.15 could be attributed to deprotonation of the carboxyl group^49^. After the pH value exceeded 9.00, the second proteolytic equilibria was established. The pKa_2_ value equalling 9.16 corresponded to deprotonation of the epsilon amine moiety on the polymer side chain. Moreover, in strong alkaline conditions (pH > 10.50), the third deprotonation step of poly-l-lysine with a pKa_3_ value equalling 10.67 was observed as a results of the loss of a proton of the alpha amine group. Due to the above, the PLL linker should be positively charged under physiological conditions as a consequence of the protonated amine group in the polymer side chains. As a results, strong electrostatic interactions are formed between the cationic PLL linker and the negative charge on the deprotonated P_x_O_y_ group on few layer black phosphorus. Moreover, the adheration of the PLL-peptides hybrid to the FLBP surface could be stabilised by hydrophobic force. During this process, the non-polar butyl chains of the poly-l-lysine are exposed for interaction with the few-layer black phosphorus nanomaterial. The non-covalent binding modes, including the synergetic effect of hydrophobic and electrostatic forces, contributed to the FLBP-PLL-peptides’ strong assembly. The efficiency of the conjugation and nanoparticles’ functionalisation were confirmed by transmission electron microscopy (TEM), scanning electron microscopy (SEM) and spectroscopic techniques (Raman and FT-IR).

The application of the covalent immobilisation protocol to coupling the PLL linker with the peptide allowed a stronger bond and therefore a more stable hybrid to form than physisorption. The biocompatible FLBP-PLL-peptide conjugates were synthesised according to the proposed non-covalent strategy. The attachment of the cationic linker with the peptide to the surface of the black phosphorus was stabilised by electrostatic and hydrophobic interaction. The proposed non-covalent functionalisation of the FLBP surface by poly-l-lysine improved the nanomaterial stability. The partial surface oxidation of the FLBP could even improve the properties of the FLBP-PLL. Application of RP-HPLC analysis for preliminary screening of covalent conjugation of the linker and peptide proved to be an efficient and practical method to be used in further investigations.

#### FLBP functionalisation with PEG-peptide conjugate

**Figure 4:**
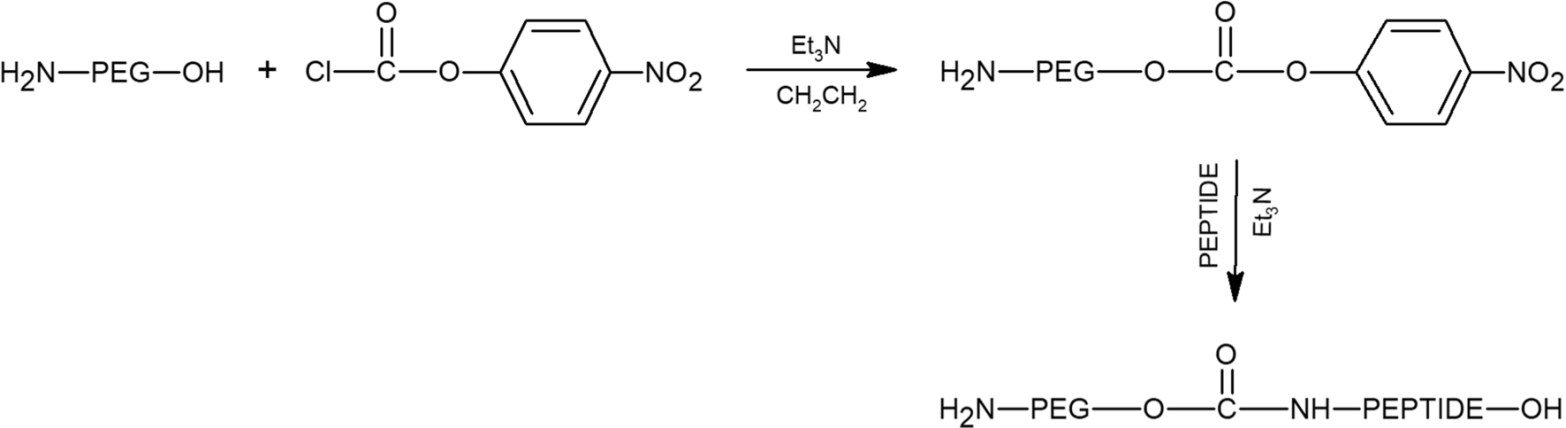
Linker (hydroxy terminated PEG) is activated to PEG-p-nitrophenyl carbonate. In the next step, peptides are PEGylated by reacting with PEG-p-nitrophenyl carbonate and produce PEG-peptide conjugates.

The FLBP surface was modified with a positively charged anchor formed the polyethylene glycol-amine-peptide connection (H_2_N-PEG-peptide) via electrostatic adsorption, and presents the dangling PEG-peptide chains to the aqueous environment. It is well established that surface coating effectively enhances their biocompatibility and physiological stability. In order to obtain PEG-peptide covalent conjugation, we applied a methodology for site-specific peptide PEGylation. In the first step, hydroxy-terminated PEG (H_2_N-PEG-OH) was activated to PEG-*p*-nitrophenyl carbonate using a chloroformate activation reaction. Afterwards, the peptides were PEGylated by reacting the *N*-terminal serine residue with PEG-*p*-nitrophenyl carbonate, to produce a stable carbamate linkage between the PEG and peptide (Fig. 4).

**Figure 5:**
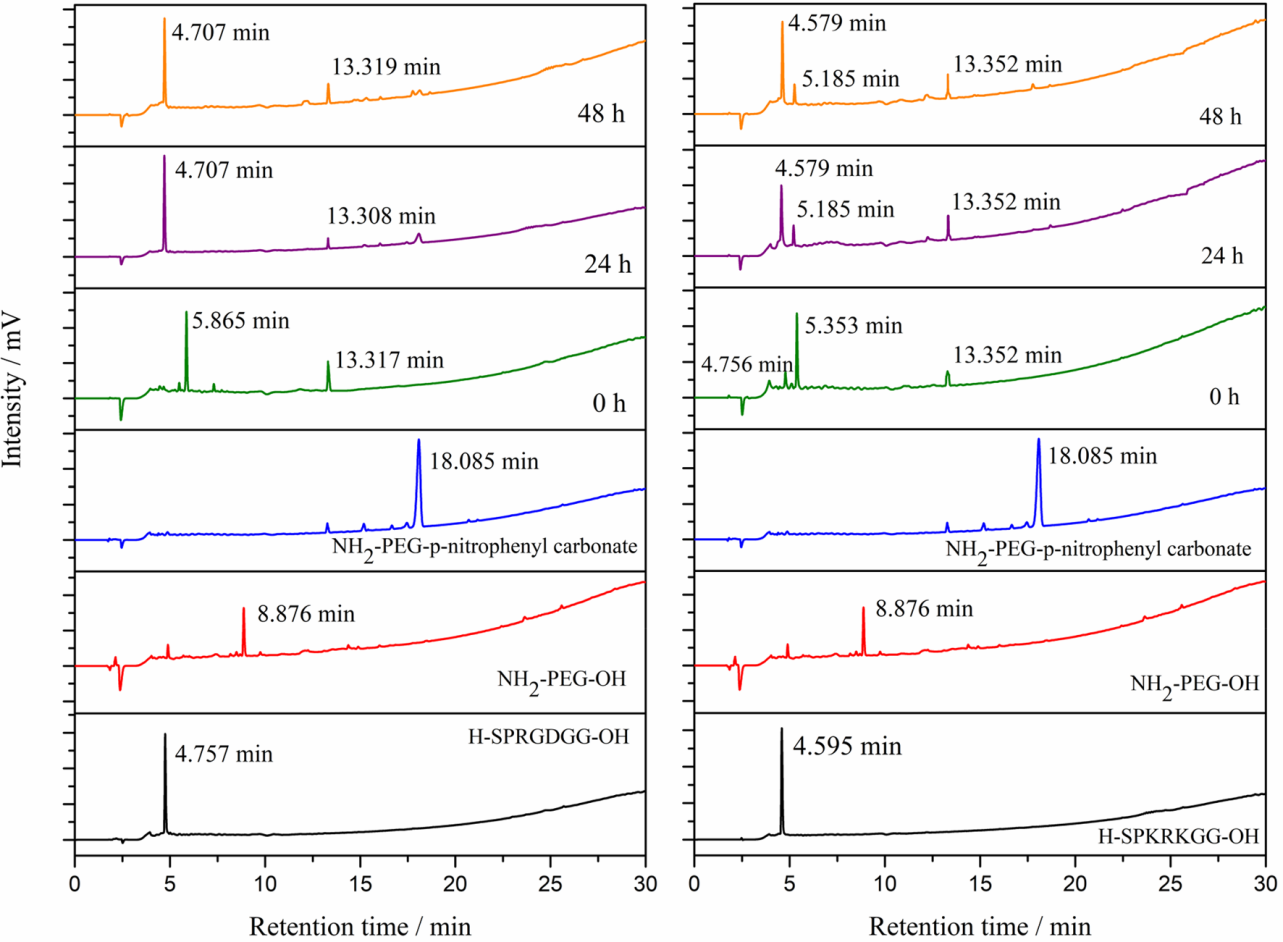
Covalent bioconjugation of PEG linker with RGD and KRK peptides’ reaction progress monitored by Reversed Phase High Performance Liquid Chromatography. RP-HPLC conditions: HPLC Shimadzu system (Shimadzu Prominence-i LC-2030C) with Cosmosil C18 column (4.60 mm × 250 mm, 90 Å, 5 µm) using linear gradient method from 0 to 100% solvent B for 30 min at a flow rate of 1.50 mL min^−1^ with UV detection at λ_1_ = 224 nm and λ_2_ = 254 nm.

The PEG-peptide conjugates were characterised by HPLC as well as MALDI-TOF mass spectrometry. The progress of the formation of the PEG-peptide conjugates was monitored by HPLC analysis of the reaction mixture at different time intervals (0, 24 and 48 h). The HPLC chromatograms of the individual substrates showed a main peak with the respective retention times of RGD, 4.757 min.; KRK, 4.595 min.; H_2_N-PEG-OH, 8.876 min.; and H_2_N-PEG-*p*-nitrophenyl carbonate, 18.085 min. (Fig. 5). The degree of completion of attachment of the PEG with the RGD and KRK peptide, respectively, indicated in both cases that the reaction was complete within 24 h. After this time, there were no significant changes in the course of the reaction, The chromatograms of the obtained bioconjugates of H_2_N-PEG-RGD-OH and H_2_N-PEG-KRK-OH demonstrate the main product at retention times of 4.707 and 4.579 min, respectively (Fig. 5).

**Figure 6:**
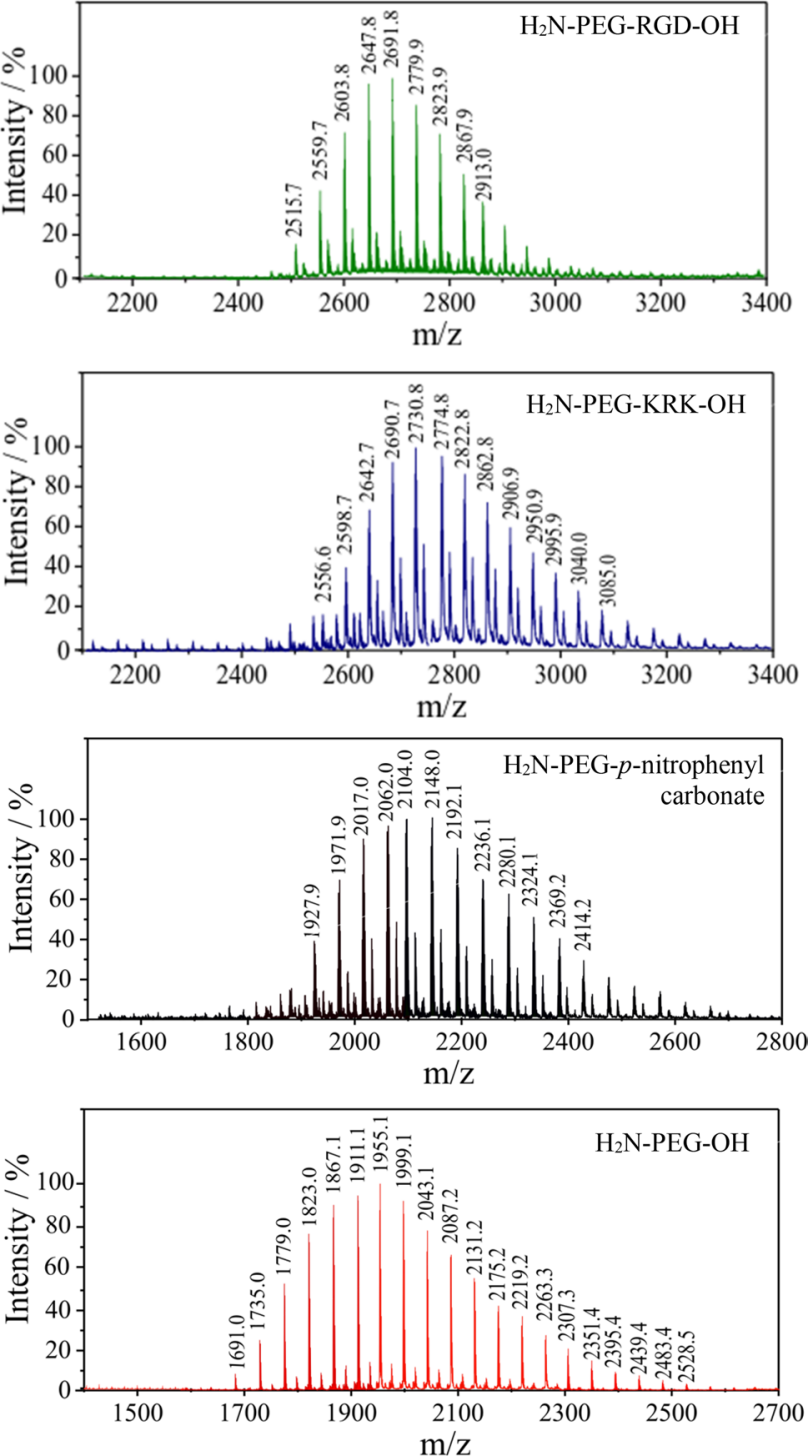
MALDI-TOF mass spectra of: native H_2_N-PEG-OH polymer, activated H_2_N-PEG-*p*-nitrophenyl carbonate, H_2_N-PEG-RGD-OH conjugate, H_2_N-PEG-KRK-OH conjugate.

The MALDI-TOF mass spectra of the native H_2_N-PEG-OH, activated H_2_N-PEG-*p*-nitrophenyl carbonate, as well as the H_2_N-PEG-RGD-OH and H_2_N-PEG-KRK-OH conjugates are shown in Fig. 6. The PEG mass spectra are characterised by a series of peaks where the mass difference between each adjacent peak is 44 mass units, which corresponds to the mass of a single ethylene glycol repeat unit and confirms that the analyte is the PEG polymer. The spectrum of the H_2_N-PEG-OH 2000 contains oligomer peaks extending from approximately m/z 1735 to 2660. The number-average the molecular weight of the H_2_N-PEG-OH 2000 is 1955.1 Da. Analysis of the peak at m/z = 1955,1 reveals that this peak is a PEG with 44 repeat ethylene glycol units. The average MW measured for the H_2_N-PEG-*p*-nitrophenyl carbonate (2150 Da) is in good agreement with the theoretical value (2134 Da). The spectrum of the H_2_N-PEG-*p*-nitrophenyl carbonate compared to the native H_2_N-PEG-OH spectrum resulted in the whole mass distribution curves being shifted to higher molecular weights while the shape of the curves show no significant changes, indicating a complete coupling reaction.

After the attachment of the RGD peptide, the number average molecular weight of the H_2_N-PEG-RGD-OH connection is expected to be 2612.6 Da. The experimental mass (2603.9 Da) is well within the expected range. The calculated mass of the H_2_N-PEG-KRK-OH conjugate (2696.8 Da) is in accordance with the observed mass (2690.7 Da). For both the H_2_N-PEG-RGD-OH and H_2_N-PEG-KRK-OH conjugates, the experimental MALDI-TOF MS results are in good agreement with the calculated values. The deviation between the calculated and the experimental masses can be neglected.

PEG was chosen as a linker for two reasons. First, it protects the FLBP structure from outer water, oxygen and non-specific interior interactions, and increases the stability of FLBP in physiological conditions. Oxidised FLBP is not stable with PEG as indicated by positive adsorption energy values. Furthermore, the use of a non-ionic and hydrophilic linker can overcome the problem of nonspecific adsorption on the surface, e.g., by proteins from blood serum or a bioanalyte sample. Secondly, a stable immobilisation of peptides on a PEG linker prolongs circulation in the blood and prevents drug release during its transport, which may improve the pharmaceutical properties of the peptides.

### Ab-initio characterisation of FLBP surface with PEG and PLL

**Figure 7:**
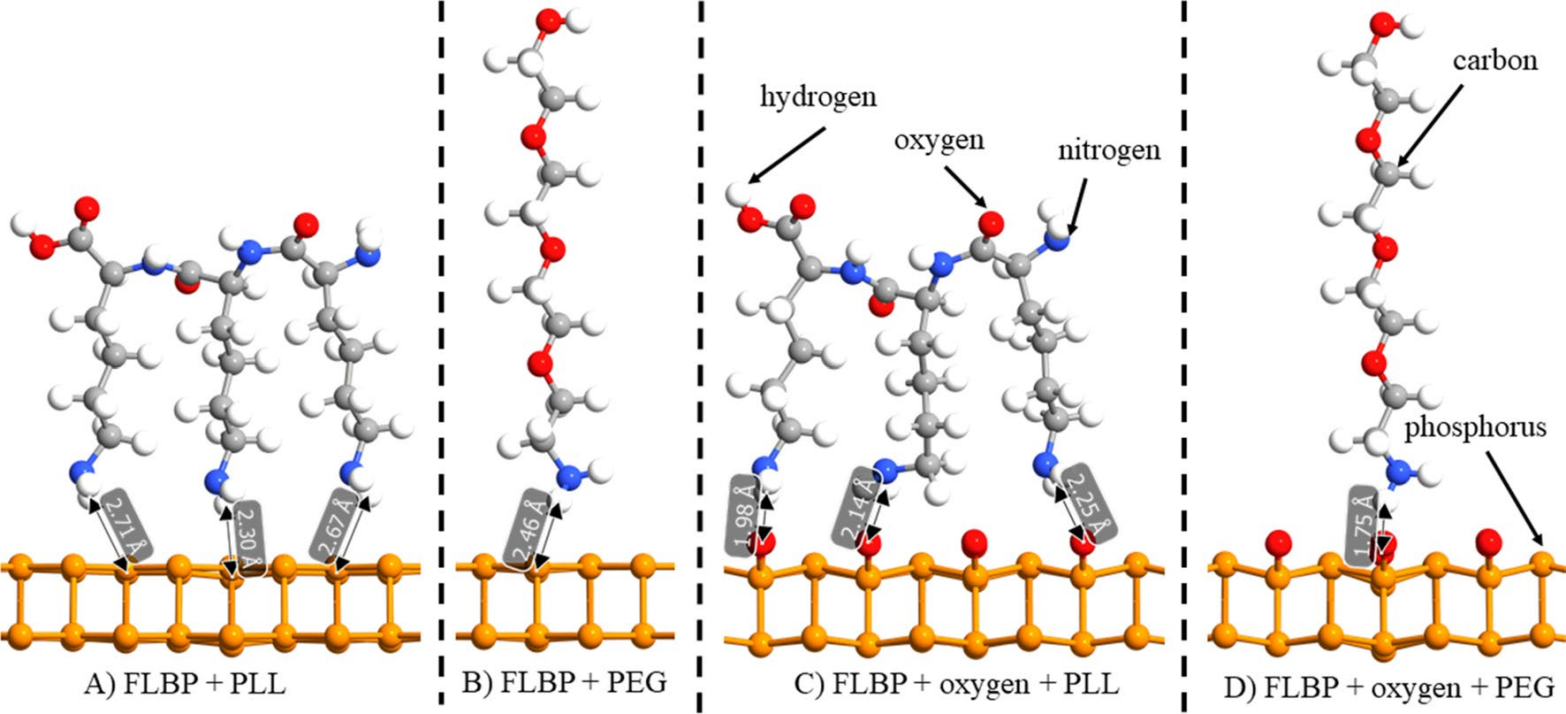
Oxidised and non-oxidised surfaces of FLBP with different linkers: (**a**) non-oxidised with PLL, (**b**) non-oxidised with PEG, (**c**) oxidised with PLL and (**d**) oxidised with PEG as linker [drawn with QuantumATK Q-2019.12 (https://www.synopsys.com/silicon/quantumatk/resources/release-notes.html)].

The four layer model (two shown in Fig. 7) of the FLBP surface was elaborated as an optimal configuration to simulate the linker grafting efficiency and stability. The FLBP bandgap predicted with DFT was in agreement with prior literature results (0.25 eV)^50,51^. The PLL and PEG polymer linker bonding was simulated utilizing estimation of absorption energy on ideal pristine and defected oxidized FLBP (see Fig. 7). In order to simulate the atmospheric degradation of FLBP, we have saturated 50% of its surface with oxygen atoms as displayed in Fig. 7c,d. The simulated adsorption energies predicted for PLL and PEG are listed in Table 1.

**Table 1:** Results of simulation of adsorption energy for pristine FLBP and oxidised FLBP with PLL and PEG as linkers.

Substrate	PLL (kcal mol^−1^)	PEG (kcal mol^−1^)
Pristine FLBP	− 3.92	− 6.89
FLBP + oxygen	− 9.34	13.94

The PLL molecule adsorption on pristine FLBP shows lower stability revealing the smallest adsorption energies required for its attachment. The attachment of PEG results in almost doubled values of adsorption energy in comparison to PLL. This fact is attributed to relatively large partial positive charge of the PEG chain amine group^52^. Simulations involving PEG adsorption display also relatively lower distances to the FLBP surface due to specific lateral stereochemical elongation. Moreover, the defected FLBP regions reveals much smaller distances to both PLL and PEG molecules since the intensive electrostatic interactions are induced by deprotonated P_x_O_y_ group simulated here as surficial oxygen. Nevertheless, the cationic PLL linker results also in a much larger negative adsorption energies caused by FLBP surface oxidation. It should be noted that adsorption of PEG at oxidized FLBP switches energies to large positive values suggesting suppression of positively charged PEG from a stable adsorption. Achieved results suggest that the polymer adsorption process would possess heterogenous behavior depending strongly on the FLBP surface quality, while it could be conducted effectively in the oxygen-free conditions.

*Ab-initio* simulation allows to conclude that PEG conjugates are more stable at the pristine FLBP than PLL conjugates as evidenced by a more negative adsorption value. Nonetheless, if the FLBP will be partially oxidized, PEG conjugates may not be effectively formed due to observed positive adsorption energy. This effect does not influence the FLBP-PLL-peptide procedure as the adsorption energy at oxidized FLBP is even more negative and generates more robust structure than pristine FLBP-PLL-peptide.

### Characterisation of structure and morphology of conjugates

#### TEM analysis

**Figure 8:**
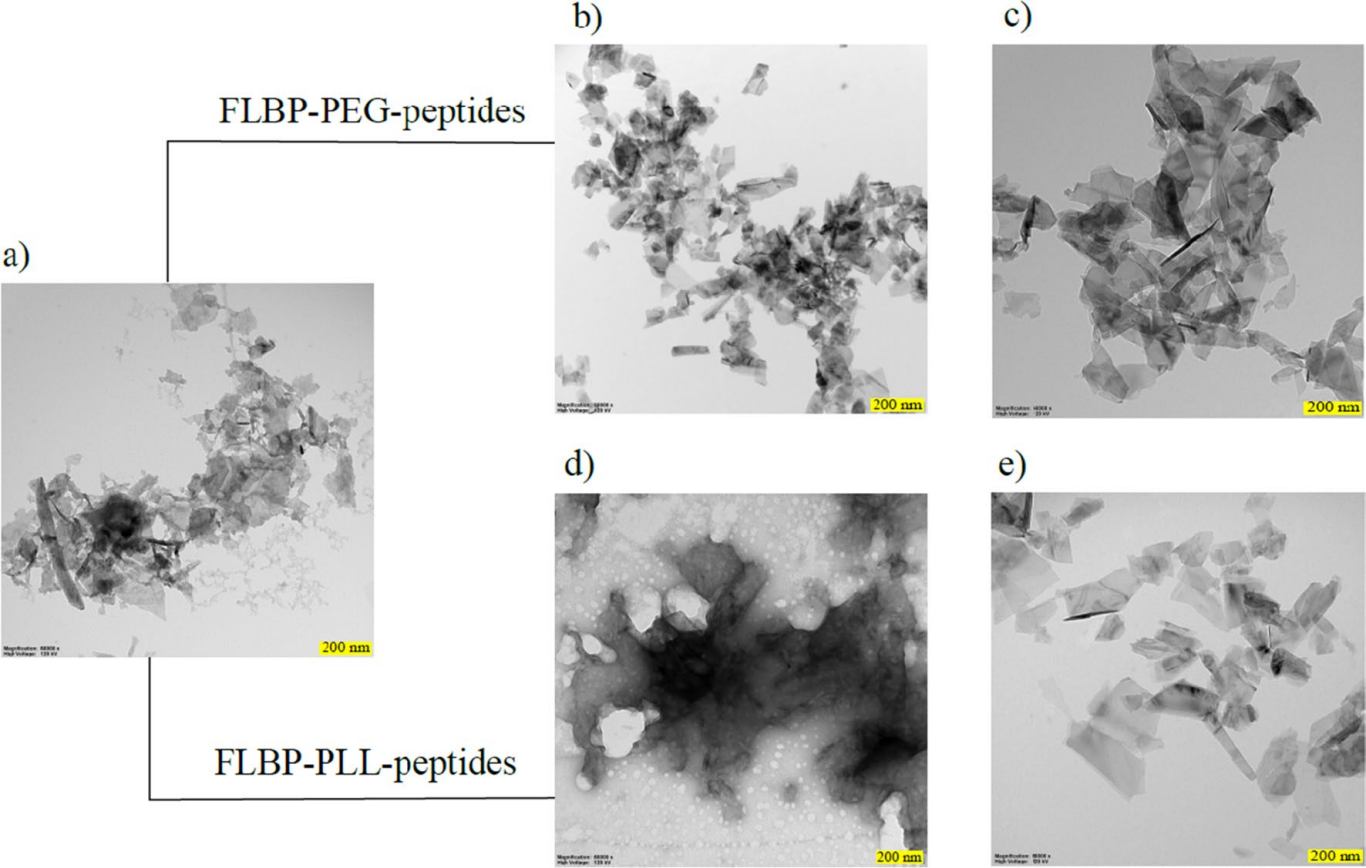
Transmission electron microscopy of exfoliated and functionalised black phosphorus nanoparticles: (**a**) FLBP, (**b**) FLBP-PEG-KRK, (**c**) BP-PEG-RGD, (**d**) FLBP-PLL-KRK, (**e**) FLBP-PLL-RGD.

The morphology of the obtained particulates was characterised by transmission electron microscopy (TEM) to quantify the shape, size and thickness. Representative TEM images of the samples (Fig. 8) revealed the presence of few-layered thin phosphorus flakes where the lateral size of the FLBP conjugates was around 200 nm. Additionally, this morphology did not show any visible degradation effects of the FLBP.

#### SEM

**Figure 9:**
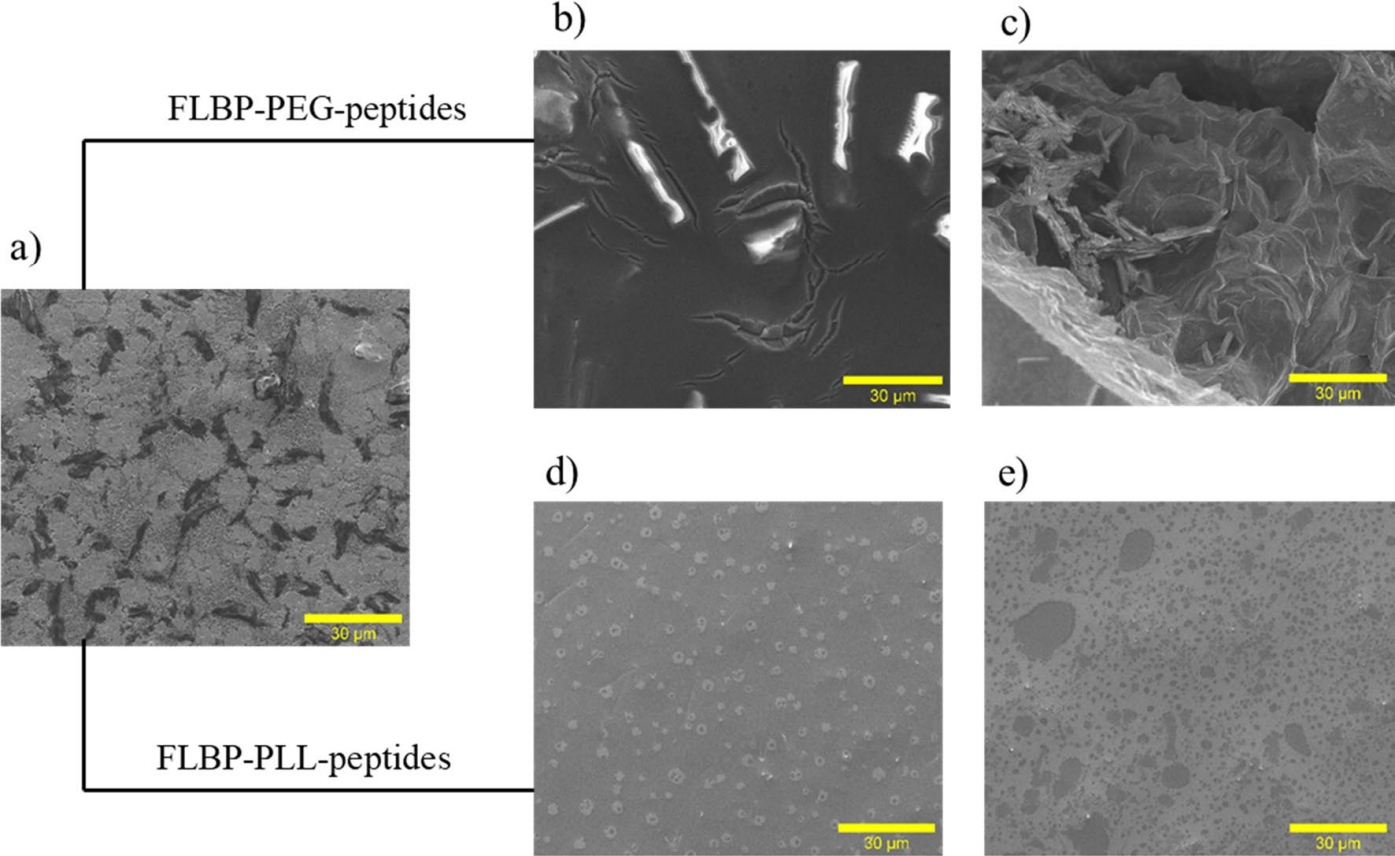
Scanning electron microscopy before and after functionalisation of FLBP: (**a**) FLBP, (**b**) FLBP-PEG-KRK, (**c**) FLBP-PEG-RGD, (**d**) FLBP-PLL-KRK, (**e**) FLBP-PLL-RGD.

SEM micrographs demonstrate that after functionalisation of FLBP by both polymers, the conjugates showed different morphologies than pristine FLBP (Fig. 9a). However, the surfaces of the FLBPs functionalised with PLL present a particle FLBP coated by PLL and peptide (Fig. 9d,e). SEM micrographs of the FLBP after PEGylation indicate a compact surface with visible bonded layers. These findings show that the structural differences depend on the linkers (PEG, PLL). The influence of the linkers is due to their structure and particularly to their charge. The non-ionic linker (PEG) forms interconnected layers (Fig. 9b,c), while the positively charged (PLL) repels the reaction products from each other and causes dispersion of the conjugates (Fig. 9d,e).

#### Infrared analysis

**Figure 10:**
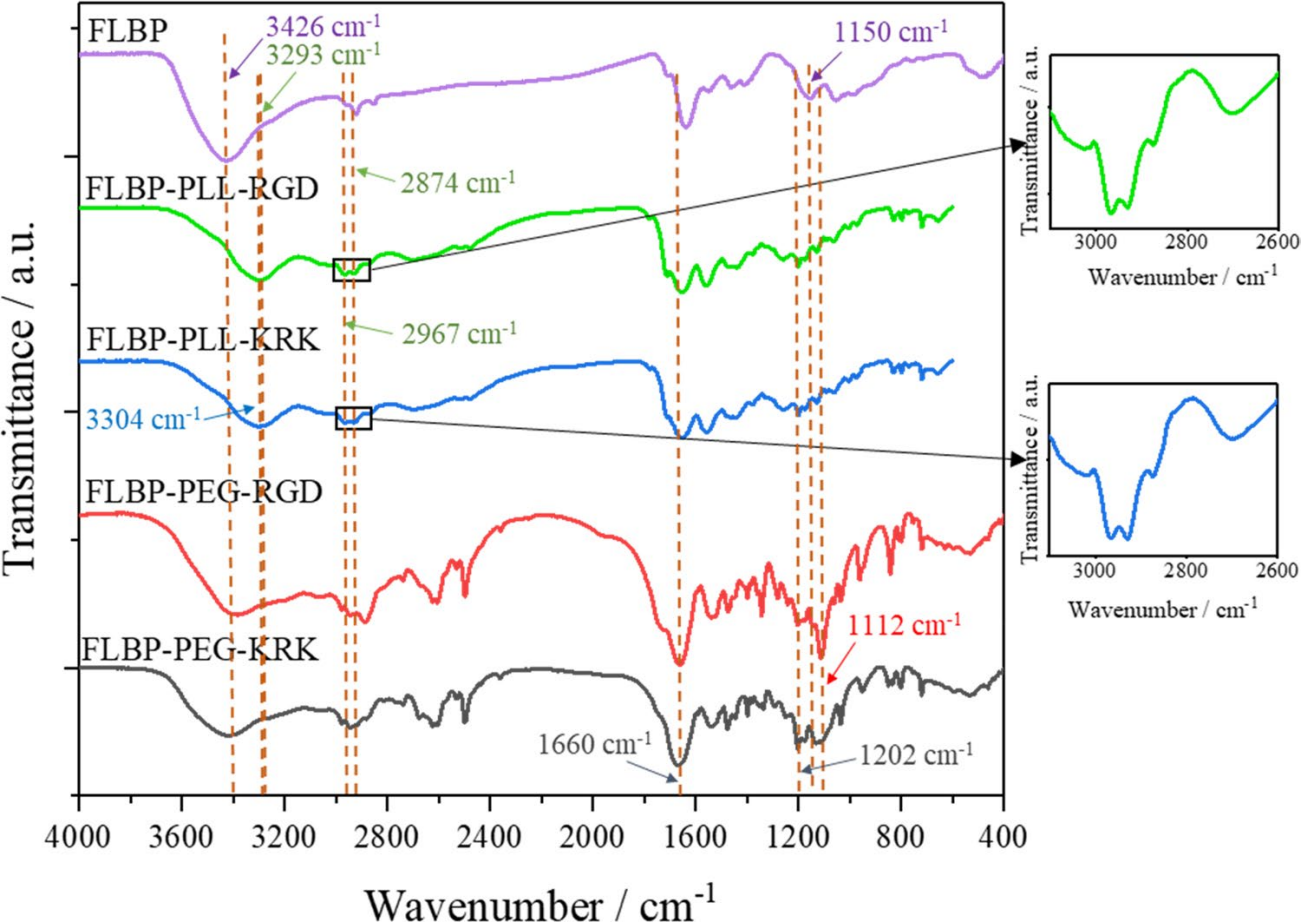
Fourier-transform infrared (FTIR) spectra for few layer black phosphorus (FLBP) and its conjugates: FLBP-PLL-RGD, FLBP-PLL-KRK, FLBP-PEG-RGD and FLBP-PEG-KRK.

Infrared spectra confirm the formation of conjugates of the FLBP with the PLL or PEG and the peptides. Figure 10 shows the spectra of the bare FLBP as well as the FLBP-PLL-peptide and FLBP-PEG-peptide. Fourier transform infrared spectroscopy (FTIR) analysis was acquired from 4000 to 400 cm^−1^. The FTIR spectrum of the FLBP-PEG-peptide is characterised by a number of characteristic bands occurring in the ranges 3600–3300 cm^−1^, 3100–2200 cm^−1^, 1750–1550 cm^−1^, 1550–1300 cm^−1^, and 1300–900 cm^−1^. The broad peak observed in the FLBP sample, and for the PEG conjugates at about 3400 cm^−1^ is attributed to the –O–H stretching band, whereas the signal at about 1650 cm^−1^ corresponds to the –O–H bending mode. It can be assumed that the black phosphorus contained moisture. In the case of the PEG conjugates, this peak is shifted towards lower frequencies, and it may originate from hydroxyl stretching vibration bands in the polymer.

For the FLBP and all conjugates, a transmittance peak was recorded at approximately 1150 cm^−1^ resulting from phosphorus oxidised during synthesis (P-O stretching). The bands at 3100–2500 cm^−1^ and 1550–1300 cm^−1^ correspond to stretching and bending vibrations of functional groups CH, CH_2_, and CH_3_. The FLBP-PEG-peptide shows the characteristic narrow and intense C–O–C absorption band at 1112 cm^−1^ in the FTIR spectrum. The peak is absent for the FLBP and FLBP-PLL-peptide conjugates. Absorption bands in the region of 1300–900 cm^−1^ correspond to the presence of amino groups. In this region, there are also overlapping signals from –P–O and C–O–C stretching vibrations. For PLL conjugates at 3304 and 3293 cm^−1^ for the KRK and RGD, respectively, there are visible peaks originating from the N–H stretching band. The bands in the range 3200–2700 cm^−1^ are attributed to the -O–H stretching of the carboxylic group which confirms the presence of PLL in the conjugate. Similar to the FLBP-PEG-peptide, the FLBP-PLL-peptide has regions at 3000–2500 cm^−1^ and 1460–1370 cm^−1^ which correspond to stretching and bending vibrations, respectively, of functional groups CH, CH_2_, and CH_3_. Absorption bands in the region 1300–950 cm^−1^ correspond to the presence of amino groups.

The two regions corresponding to the presence of the peptides are the amide peaks. The more intense amide band (1600–1700 cm^−1^) is associated mainly with C=O stretching vibrations with major contributions from C–N stretching. The less intense amide band (1480–1580 cm^−1^) originates from bending motions of the N–H groups and stretching of the C–N bonds of the peptide bonds. The other amide bands are less commonly used for peptide analysis. The problem in the spectral analysis of the peptides is that the widths of the various contributing bands are usually much greater than the peak separations^53^.

#### Raman analysis

**Figure 11:**
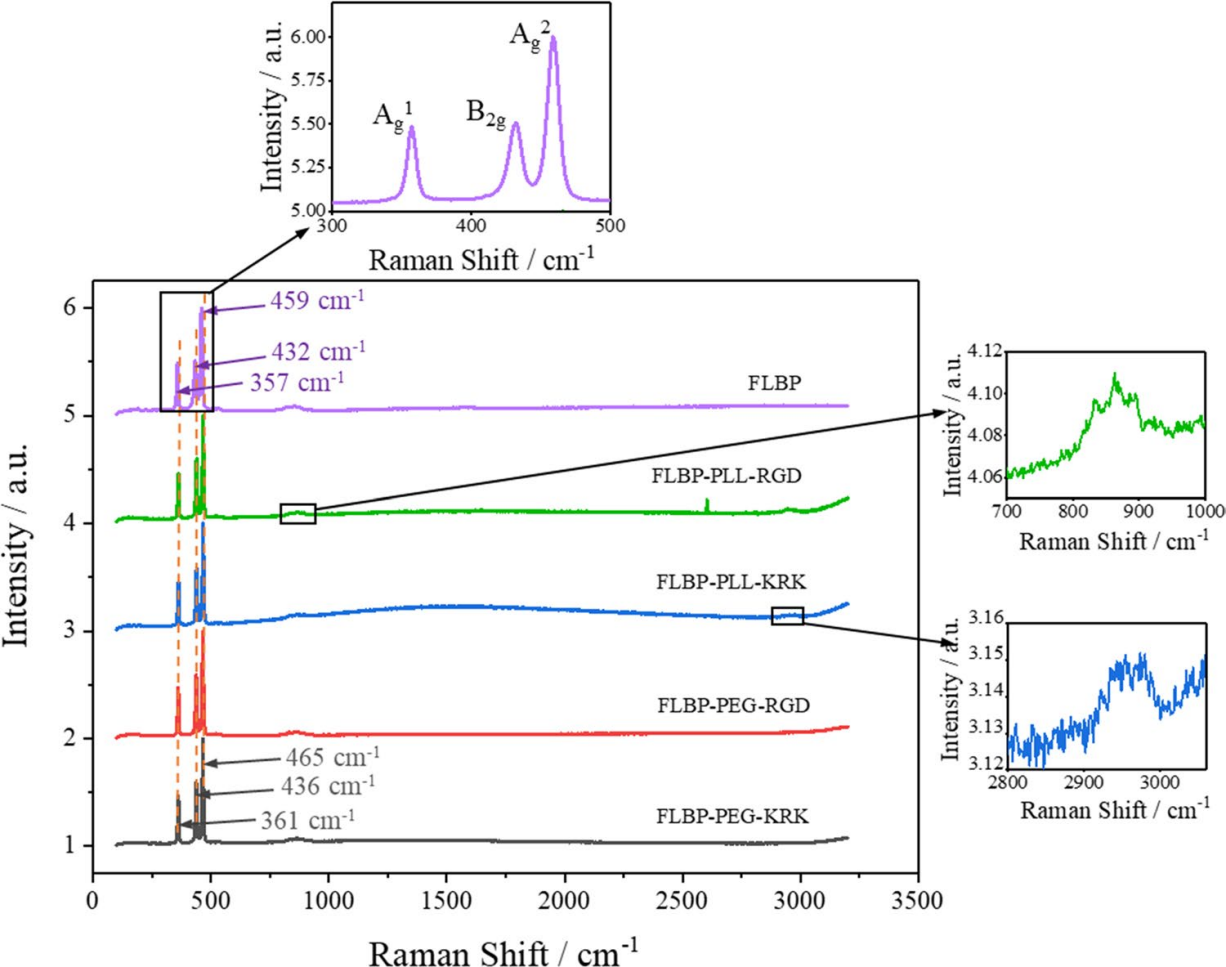
Raman spectra for few layer black phosphorus (FLBP) and its conjugates: FLBP-PLL-RGD, FLBP-PLL-KRK, FLBP-PEG-RGD and FLBP-PEG-KRK.

In Fig. 11, the Raman spectra confirms the presence of black phosphorus in each tested conjugate. We have shown that a typical characteristic for few-layer black phosphorus is maxima near 357, 432, and 459 cm^−1^ for bare FLBP corresponding to the A_g_^1^, B_2g_ and A_g_^2^ modes, respectively. The relative band intensities in the Raman spectra recorded for the conjugates were shifted to 361, 436 and 465 cm^−1^ for the A_g_^1^, B_2g_,and A_g_^2^ modes, respectively, which is related to the additional stresses associated with the connection of the FLBP surface with the polymer and peptide. The ratio of the intensity of the A^1^_g_ and A^2^_g_ vibrational modes was applied to study FLBP stability. The subsequential measurements of pristine FLBP and two-week aged FLBP conjugates revealed similar values of A^1^_g_/A^2^_g_ ratios proving minor changes in the amount of oxygen on the FLBP-PLL-peptide and FLBP-PEG-peptide surfaces^54,55^. This confirms the stability of the FLBP conjugates.

In the range 800–900 cm^−1^, abnormal peaks can be noticed. This is associated with the anisotropy of FLBP, and the literature shows a strong dependence on the azimuthal angle. It has been proven by Xi Ling et al. that these the newly anomalous phonon modes are caused by the resonant Raman effect^56^. The carbon-hydrogen stretching region ranges from 2800 to 3000 cm^−1^ and includes a series of overlapping vibrational bands correlated with the acyl group and overtones of the methylene deformation modes. The peaks are attributed to the methylene symmetric and asymmetric C-H stretching vibrations^57^.

### Cytotoxicity of FLBP functionalisations

**Figure 12:**
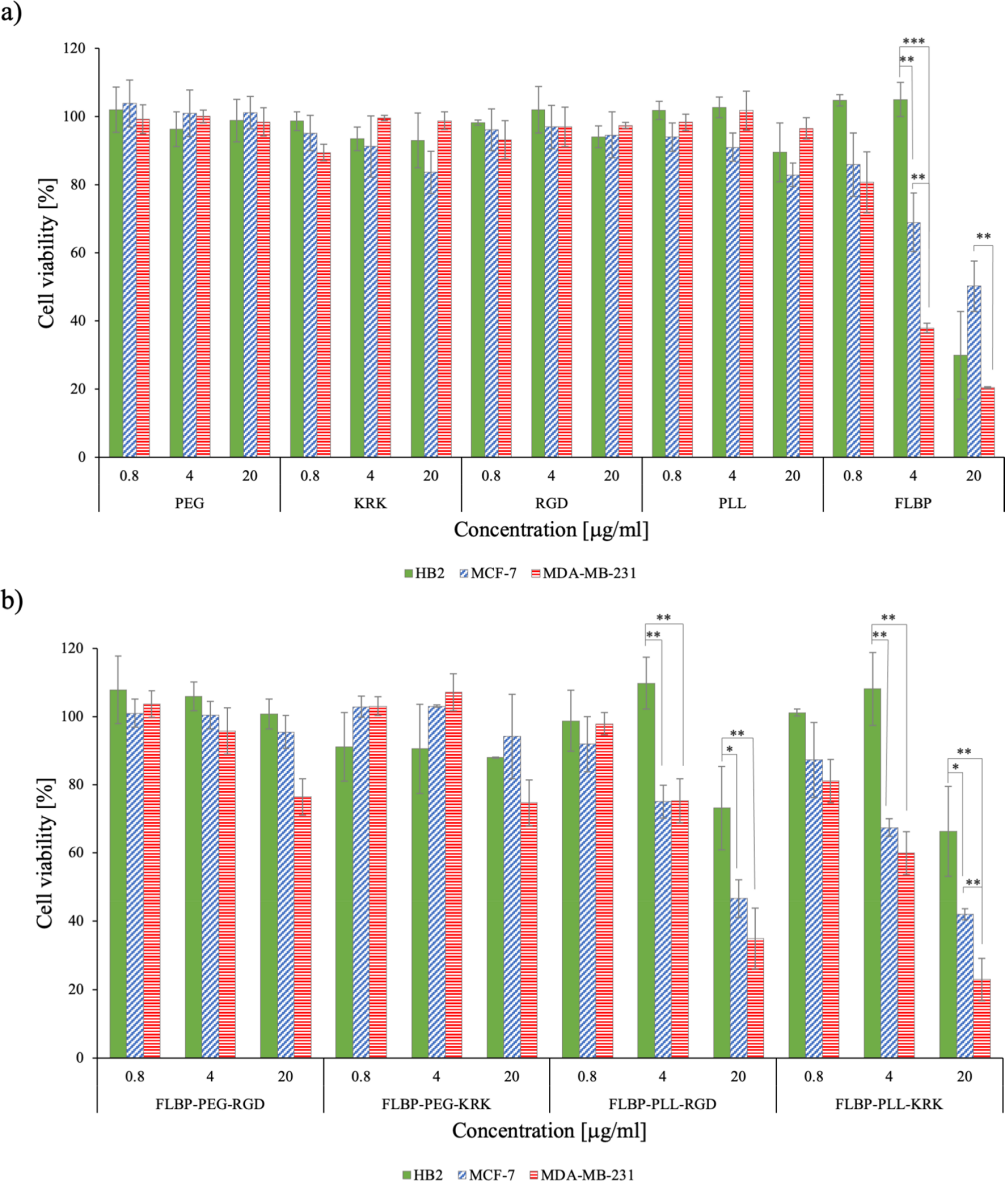
Cell viability [%] of three different cell lines HB2, MCF-7 and MDA-MB-231 after 72 h incubation with various concentrations (0.8, 4, 20 μg ml^−1^) of (**a**) PEG, KRK, RGD, PLL, FLBP and (**b**) FLBP-PEG-RGD, FLBP-PEG-KRK, FLBP-PLL-RGD, FLBP-PLL-KRK determined by MTT assay. Data are normalised to control (cells incubated without any additives [0 μg ml^−1^]) and presented as means ± SD (n = 3; Student’s t-test; *P < 0.05, **P < 0.01, ***P < 0.001).

To investigate the cytotoxic effects of FLBP and its modifications on breast cancer cell lines, MCF-7, MDA-MB-231 and HB2 cells were treated with increasing concentrations of PEG, KRK, RGD, PLL, FLBP, BP-PEG-RGD, BP-PEG-KRK, BP-PLL-RGD, and BP-PLL-KRK (0, 0.8, 4, 20 mg ml^−1^) for 72 h. The MTT assay revealed cell- and dose-dependent cytotoxicity of FLBP and its modifications. HB2 (normal mammary cells) was affected only at the highest concentration of FLBP (20 μg ml^−1^), whereas the viability of the MCF-7 and MDA-MB-231 breast cancer cells decreased at 4 μg ml^−1^. Comparing these two cancer cell lines, FLBP at a concentration of 4 μg ml^−1^ was more toxic to the aggressive cell line, MDA-MB-231 (38% viability), than to MCF-7 (69% viability). No components of the subsequent FLBP modifications showed toxicity (Fig. 12a). Modifications of FLBP with PEG-peptide seems to completely neutralise FLBP’s toxicity in all tested cells. Even at the highest concentration, cell viability was more than 80% (Fig. 12b). Attaching PLL-peptide to FLBP resulted in reduced cytotoxicity, but only in normal mammary cells; HB2 cells exhibited ~ 70% cell viability at 20 μg ml^−1^, while the cancerous cells were found to be more sensitive to this modification. In addition, this modification is more toxic to the triple-negative breast cancer cell culture—MDA-MB-231 (viability < 34%) compared to the luminal breast cancer cell culture—MCF7 (viability < 46%).

The biocompatibility of black phosphorus is unclear. Some studies show that BP has no cytotoxic effect on cells^17,58,59^, but none of them incubated this material with cells for longer than 48 h. A more probable hypothesis is that the toxicity of black phosphorus is dependent on the time, dose and tested cell type^59,60^, which is in agreement with our results. The cytotoxicity of FLBP is most likely due to membrane disruption and oxidative stress-mediated metabolic activity reduction^18,61^. Recently, it was shown that BP-based materials can induce higher levels of reactive oxygen species (ROS) in cancer cells, leading to significant changes in the cytoskeleton, DNA damage, G2/M arrest and apoptosis. It may be due to decreased superoxide dismutase (SOD) activity by lipid peroxides in cancer cells^62^. The cellular internalization and degradation of BP-based nanomaterials is at higher rate in cancer cells what increases level of phosphate anions and induced high ROS production. Those selecting cytotoxic effect occurred only within certain concentration. High dosage of BP-based compounds was toxic also to normal cells by inducing high ROS production.”

The biocompatibility and stability of BP may be improved by its modification and packaging^10,63^. PEG, which has gained FDA approval, has been widely used in various biomedical fields due to its biocompatibility and hydrophilicity, it was shown that PEGylation can improve water-stability of drugs^64^. Here we observed that conjugation of PEG-peptide abolished the toxicity in all tested cells, suggesting that this modification could be implemented as a therapeutic delivery carrier. Conjugates FLBP-PLL-peptide was also neutral for normal mammary cells but exhibited strong toxicity to breast cancer cell lines. It could have been due to the fact that PLL, cationic polymer, has slightly worse biocompatibility than PEG, it can induce apoptosis by damaging cell membrane^65,66^.

It is worth nothing that FLBP is not significantly cytotoxic at concentration lower than 4 μg mL^−1^, suggesting that FLBP in the range of only few μg mL^−1^ can be effectively used in biomedical applications. It has been revealed that the functionalisation of FLBP with PLL allows not only the enhancement of biocompatibility, but also suppress the degradation into nontoxic phosphate and phosphonate. These results showed that, although the cytotoxicity of FLBP is closely dependent on their concentration, the FLBP with the desirable functionalisation can be compatibly employed in biomedical applications, even at concentration higher than 4 μg mL^−1^. It also showed that presented conjugates could be applied as delivery platform where concentration of drug should be higher than 4 μg mL^−1^. Taken these results together, it is concluded that the reduced toxicity of tested conjugates against normal cell was attributed to KRK and RGD peptides, which could bond to the receptor specifically.

Our results show that PLL itself is neutral for all tested cells but toxicity of PLL conjugated with KRK and RGD is cancer cells specific. This suggests that KRK and RGD motifs have great potential for targeting tumor cells and applying FLBP-PLL-peptide for cancer treatments could enhance the cytotoxic effect of cancer drugs.

## Conclusions

Summarizing, we have successfully conducted two novel functionalisation procedures resulting in fabrication of FLBP-PEG and FLBP-PLL tumor-homing peptide conjugates. PEG and PLL were critically compared as a spacer molecules due to their different polymer masses, structures and cytotoxic activity. The effects were demonstrated using in vitro experiments.

Both TEM and SEM micrographs demonstrated shaped typical for functionalised FLBP by polymers revealing conjugates of 200 nm with in contrast to pristine FLBP. Raman proved the stability of functionalised FLBP, while FT-IR studies confirmed both spacer and peptides grafting.

The experimental data were supported by *ab-initio* model manifesting that PEG-peptide adsorption is favored at the pristine FLBP. Interestingly, the partially oxidized FLBP still allows for an efficient PLL-peptide adsorption in contrast the suppression of PEG molecules due to the deprotonated P_x_O_y_ surficial groups. Data showed that polymer adsorption process would reveal specific heterogenous behavior depending on the FLBP surface termination.

FLBP exhibited different levels of toxicity depending on its concentration, type of modification and the tested cell line. Functionalisation of FLBP with PLL reduced cytotoxicity in normal mammary cells but was toxic to cancerous cells, especially to the triple-negative breast cancer cell culture—MDA-MB-231. These findings suggest that applying FLBP-PLL as a therapeutic delivery carrier for cancer treatments could enhance the cytotoxic effect of cancer drugs, although further comprehensive studies are necessary to understand the mechanism of its toxic effects. Proposed functionalisation by decoration surface of FLBP with polymeric micelles allowed to tune its biocompatibility, degradability and drug loading ability for controlled and targeted anticancer drug delivery therapies. The non-ionic and hydrophilic linkers overcome the problem of non-specific adsorption on the FLBP surface, e.g., by proteins from blood serum or a bioanalyte sample. Next, the stable immobilisation of peptides on a PEG linker prolongs circulation in the blood and prevents drug release during its transport.
